# Influence of socioeconomic inequality on the distribution of COVID-19 hospitalizations and deaths in Brazilian municipalities, 2020: an ecological study

**DOI:** 10.1590/S2237-96222023000100021

**Published:** 2023-02-10

**Authors:** Gabriela Drummond Marques da Silva, Anelise Andrade de Souza, Mônica Silva Monteiro de Castro, Wanessa Debôrtoli de Miranda, Leticia Lemos Jardim, Rômulo Paes de Sousa

**Affiliations:** 1 Fundação Oswaldo Cruz, Instituto René Rachou, Belo Horizonte, MG, Brazil; 2 Universidade Federal de Ouro Preto, Escola de Nutrição, Ouro Preto, MG, Brazil; 3 Universidade Federal de Minas Gerais, Escola de Enfermagem, Belo Horizonte, MG, Brazil

**Keywords:** Coronavirus, Hospitalization, Mortality, Socioeconomic Factors, Ecological Study

## Abstract

**Objective:**

to analyze the influence of socioeconomic inequality on COVID-19 distribution in larger Brazilian municipalities, controlling for effect of hospital infrastructure, comorbidities and other variables.

**Methods:**

this was an ecological study of COVID-19 hospitalizations and deaths in 2020; outcome data were obtained from the Ministry of Health; incidence ratios were estimated using a generalized linear model.

**Results:**

we identified 291,073 hospitalizations and 139,953 deaths; we found higher mortality rates in municipalities with a higher proportion of non-White people (95%CI 1.01;1.16) and with more households with more than two people per room (95%CI 1.01;1.13); presence of sewerage systems was protective for both outcomes (hospitalizations: 95%CI 0.87;0.99 – deaths: 95%CI 0.90;0.99), while a higher proportion of the population in subnormal housing clusters was a risk factor (hospitalizations: 95%CI 1.01;1.16 – deaths: 95%CI 1.09;1.21), with this variable interacting with the proportion of people receiving Emergency Aid (hospitalizations: 95%CI 0.88;1.00 – deaths: 95%CI 0.89;0.98).

**Conclusion:**

socioeconomic conditions affected illness and death due to COVID-19 in Brazil.

## INTRODUCTION

The first cases of COVID-19 were reported in China. The disease quickly spread across the globe, leading to a pandemic, as declared by the World Health Organization (WHO) in March 2020. That year, more than 82.5 million cases and approximately 1.8 million deaths due to COVID-19 were confirmed. Of this total, the Americas accounted for 44.5% of cases and 47.45% of deaths.^1^

Brazil accounted for 9.2% of confirmed cases and 10.7% of deaths in 2020,^2^ ranking third in the world in the number of cases and second in the number of deaths, coming only after the United States.^1^ Incidence of cases, hospitalizations and deaths was heterogeneous between Brazilian states, municipalities and population groups.^3^

Restrictive measures and social distancing, such as the use of masks, restriction of people’s movements and suspension of non-essential activities, although necessary and relevant, proved to be insufficient to contain the growth in the number of COVID-19 cases in Brazil.^4^ Despite the implementation of Emergency Aid, a benefit designed to guarantee a minimum income to Brazilians in situations of greater vulnerability, the effects of the fall in economic activity worsened already existing social inequality, making it difficult for this population to adhere to the recommendation of restricted mobility^4^ and contributing to the worsening of the pandemic in the country.

**Table:** 

Study contributions	
**Main results**	The COVID-19 mortality rate was higher in municipalities with a larger non-White population, subnormal housing clusters and households with more than two persons per room, and without sewerage. Effect of interaction between subnormal housing clusters and Emergency Aid.
**Implications for services**	Addressing socioeconomic inequalities demands inclusive policies for vulnerable people, in order to favor their access to public and private goods and services essential for their health and well-being.
**Perspectives**	It is fundamental to go beyond interventions focused on single strategies in a context in which sectoral systems act in isolation, and instead invest in universal interventions that seek transversal and intersectoral articulation.

There is therefore a need to understand the factors that contributed to the greater impact of the pandemic in certain regions and population groups, so as to enable public policies to be executed more efficiently in Brazil, with the occurrence of future pandemics in mind. The objective of this study was to analyze the hypothesis that socioeconomic inequality influenced COVID-19 distribution in the largest Brazilian municipalities, while controlling for the effect of hospital infrastructure, comorbidities and other variables.

## METHODS

### Study design

This was an ecological study which analyzed COVID-19 hospitalizations and deaths recorded from March 2020, the month of the first confirmed COVID-19 death in the country, until December of the same year, in 326 Brazilian municipalities with more than 100,000 inhabitants. Selection of municipalities where 57.4% of the country’s population lives is justified by the fact that these municipalities accounted for 71.8% of the 194,949 COVID-19 deaths registered in Brazil in 2020.^2^

### Information systems used and variables analyzed

**Figure 1:**
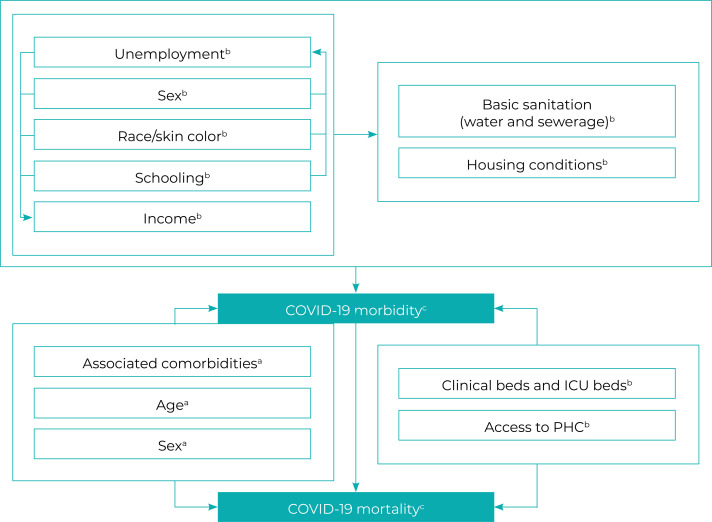
– Mechanisms linking economic and social inequalities to COVID-19 morbidityd and mortality a) Proximal determinants; b) Distal determinants; c) Outcome; d) In this study, morbidity was estimated based on COVID-19 hospitalizations. Note: ICU = intensive care unit; PHC = Primary Health Care.

Following the literature review that formed the basis for the theoretical model used, we selected the study’s independent variables, grouping them into both control variables^5^ related to hospital infrastructure and comorbidities, and also into the main socioeconomic exposure variables^10^ (Figure 1).

The dependent variables – COVID-19 hospitalizations and deaths – were obtained in June 2021. The information on COVID-19 deaths that occurred in 2020 was extracted from the Coronavirus Panel,^2^ which contains information on the pandemic in Brazil, while the information on hospitalizations in 2020 was obtained from the Hospital Information System of the Brazilian National Health System [*Sistema de Informações Hospitalares do Sistema Único de Saúde* (SIH/SUS)]. Both the Coronavirus Panel and the SIH/SUS system are maintained by the Ministry of Health.

The information related to the dependent variables was identified according to municipality of residence, date of hospitalization and date of laboratory or clinical-epidemiological confirmation of the cases. In order to calculate the COVID-19 hospitalization and death rates in the municipalities in 2020, the COVID-19 hospitalizations and deaths recorded in each location in that year (numerator) were divided by the population estimates made by the Brazilian Institute of Geography and Statistics (*Instituto Brasileiro de Geografia e Estatística* - IBGE) for each municipality for the same year (denominator), and multiplied by 100,000.

The main exposure variables, selected according to the availability of municipal socioeconomic indicators held on the databases of the IBGE and those of the Social Development Ministry, the Health Ministry and the Regional Development Ministry, were:

- IBGE – (i) proportion (%) of literate people among the population aged 15 and over in 2010, (ii) proportion (%) of non-White people in 2010, (iii) proportion (%) of people living in households with more than two people per room in 2010, (iv) Gini index of *per capita* income in 2010 (the index measures the level of income concentration and varies from 0 to 1, where 0 represents absence of inequality and 1 represents maximum inequality), (v) proportion (%) of households in subnormal housing clusters and more than a kilometer away from primary health care facilities in 2019 and (vi) proportion (%) of households in subnormal housing clusters in 2019;
- Social Development Ministry – (vii) proportion (%) of people eligible for the Emergency Aid benefit in 2020;
- Health Ministry – (viii) proportion (%) of people with access to SUS Primary Health Care in December 2018 and (ix) number of rapid RT-PCR COVID-19 tests per 100 cases (tests/100 cases) in 2020; and
- Regional Development Ministry – (x) proportion (%) of people with access to safe drinking water and (xi) sewerage system (at least basic) in 2018.

The control variables, obtained from databases made available by the Ministry of Health and also collected at the municipal level, were organized into two dimensions, according to the study hypothesis:

- Hospital infrastructure – number of SUS clinical inpatient beds per 100,000 inhab. (i) in January 2020 and (ii) in July 2020 and (iii) number of private clinical inpatient beds in January 2020 and (iv) in July 2020 (beds/100,000 inhab.);
- Comorbidities – number of hospitalizations per 10,000 inhab. in 2019, due to (v) diabetes, (vi) hypertension and (vii) chronic respiratory diseases, standardized by age (hospitalizations/10,000 inhab.), and number of deaths per 100,000 inhab. in 2019 (viii) due to cardiovascular diseases, (ix) due to diabetes *mellitus*, (x) due to malignant tumors and (xii) due to chronic respiratory diseases, standardized by age (deaths/100,000 inhab.).

In order to calculate the number of private sector clinical beds per 100,000 inhab., the total number of beds per municipality – in this sector – was divided by the population benefiting from health insurance in each municipality in December 2019, according to the National Supplementary Health Agency (*Agência Nacional de Saúde Suplementar* - ANS). The number of SUS beds per 100,000 inhab., was divided by the municipal population and subtracted from the total number of health insurance beneficiaries.

The comorbidity control variables were standardized by age, in five-year strata, using the direct method. The dependent variables were standardized using the indirect method, by age and sex, multiplying the non-standardized indicators of COVID-19 hospitalizations and deaths by a correction factor. The correction factor was calculated based on data on severe acute respiratory syndrome (SARS) associated with COVID-19 in 2020 and available via the Influenza Epidemiological Surveillance Information System (*Sistema de Informação da Vigilância Epidemiológica da Gripe* - SIVEP-Gripe). The correction factor was equal to the SARS hospitalization and death rate ratio in 2020, standardized by age and sex, divided by the unstandardized rate. The population data used for standardization, according to five-year age groups and sex, was obtained from Health Ministry population estimates.^13^

### Statistical analysis methods

Initially, we performed multiple imputation of information for variables with missing data, using the Predictive Mean Matching (PMM) method. We then standardized all the independent variables using the Z-score. Association of the independent variables with the COVID-19 hospitalization and death rates was then determined using a generalized linear regression model, assuming negative binomial distribution. Association was reported using incidence rate ratios (IRR) and respective 95% confidence intervals (95%CI).

The independent variables were selected using the hierarchical method, in two stages: (i) inclusion of the control variables in the generalized linear model, followed by (ii) the addition of the main exposure variables. In both stages, univariate analysis (single independent variable) (p-value < 0.20) and multivariable analysis (multiple independent variables) (p-value < 0.05) were performed. The backward method and the Akaike information criterion (AIC) were used as a strategy for selecting the best model. In the multivariable model, at least one infrastructure and comorbidity control variable was fixed, even if its p-value was > 0.05, in order to assess the study hypothesis. After the variables were selected, we tested the second-order multiplicative interaction of the main exposure variables that remained in the model along with Emergency Aid.

The final model was adjusted with and without imputation to identify changes in the statistical significance of the association of the selected independent variables with the outcome. Model multicollinearity was assessed using the variance inflation factor (VIF < 5). The quality of the model fit was checked by applying the deviance test. Residual analysis was performed by graphically evaluating the closeness of the residuals in normal distribution and in the scatter plot of their distribution, given the logarithm of the adjusted values. Possible influential points of influence were assessed using Cook’s distance.

The proportion of variance explained by the final models was estimated using the D^2^ statistic, an adjusted version of R^2^, which calculates the proportion of deviance explained by generalized linear models. The analyses were performed using the R software mice, MASS, car, boot and modEvA packages.

This study followed the ethical principles described in National Health Council Resolution (CNS) No. 466, dated December 12, 2012. As it only used public access secondary data with no identification of the participants’ names, the study project was exempted from submission to a Research Ethics Committee.

## RESULTS

We analyzed data on 326 municipalities, 154 (47.2%) of which were in the Southeast region, 64 (19.6%) in the Northeast region, 53 (16.3%) in the Southern region, 31 (9.5%) in the Northern region, and 24 (7.4%) in the Midwest region of the country. In 2020, 291,073 COVID-19 hospitalizations and 139,953 COVID-19 deaths were reported in these municipalities, equivalent to a rate of 238.6 hospitalizations/100,000 inhab. and 114.7 deaths/100,000 inhab. caused by the disease. The age- and sex-standardized hospitalization and mortality rates were 250.6 cases/100,000 inhab. and 122.3 deaths/100,000 inhab., respectively.

**Figure 2:**
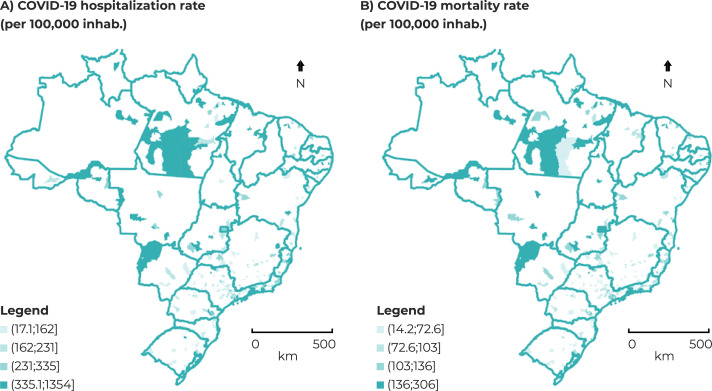
– Spatial distribution (A) of COVID-19 hospitalization rates (per 100,000 inhab.) and (B) mortality (per 100,000 inhab.), standardized indirectly by age and sex, in municipalities with more than 100,000 inhabitants, Brazil, 2020

**Figure 3:**
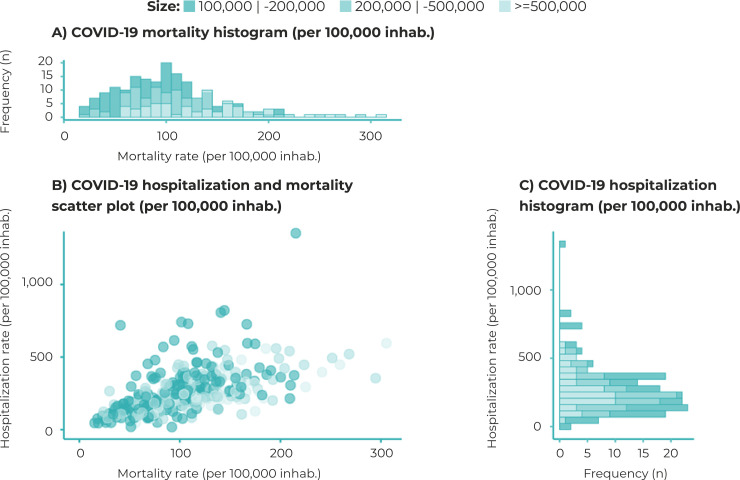
– Distribution of COVID-19 mortality rates (per 100,000 inhab.) and hospitalization rates (per 100,000 inhab.), standardized indirectly by age and sex, in municipalities with more than 100,000 inhabitants, Brazil, 2020 a) Mortality rate histogram (per 100,000 inhab.); b) Scatter plot showing hospitalization rate (per 100,000 inhab.) and mortality rate (per 100,000 inhab.); c) Hospitalization rate histogram (per 100,000 inhab.)

The majority of the municipalities with higher COVID-19 mortality rates also had higher COVID-19 hospitalization rates (Figure 2). The hospitalization rate did not show significant variation according to the size of the municipalities; a different behavior was found in the mortality rates, especially in municipalities with more than 500,000 inhabitants, which showed greater variation (Figure 3).

The socioeconomic variables, namely, (i) distance from a primary health care center, (ii) presence of subnormal housing clusters, (iii) households with more than two people per room, (iv) non-White race/skin color, (v) the Gini index and (vi) receipt of Emergency Aid, showed positive association with the hospitalization rate, while (i) the literacy rate of the population aged 15 or older, (ii) access to safe drinking water, and (iii) access to a sewerage system showed negative association with this outcome (Table 1).

**Table 1:** – Univariate analysis of outcome (COVID-19 hospitalization and mortality rates) association with the main control and exposure variables, according to a generalized linear model (GLM), in municipalities with more than 100,000 inhabitants, Brazil, 2020

Group	Variables	Hospitalization			Deaths		
		IRR^a^	95%CI^b^	p-value	IRR^a^	95%CI^b^	p-value
**Control variables**	Diabetes mellitus hospitalization rate	1.18	1.11;1.25	< 0.001	1.07	1.01;1.12	0.011
	Respiratory diseases hospitalization rate	1.14	1.08;1.21	< 0.001	1.01	0.96;1.07	0.627
	Hypertension hospitalization rate	1.13	1.06;1.22	< 0.001	1.04	0.99;1.10	0.103
	Cardiovascular diseases mortality rate	1.17	1.10;1.24	< 0.001	1.19	1.13;1.24	< 0.001
	Diabetes mellitus mortality rate	1.14	1.07;1.21	< 0.001	1.10	1.05;1.16	< 0.001
	Respiratory diseases mortality rate	1.02	0.96;1.08	0.552	1.08	1.03;1.13	0.003
	Cancer mortality rate	0.98	0.92;1.03	0.410	1.04	0.99;1.10	0.107
	Clinical beds available-SUS/100,000 inhab. (January 2020)	1.03	0.98;1.09	0.358	1.00	0.95;1.04	0.878
	Clinical beds available/private system/100,000 inhab. (January 2020)	1.06	1.00;1.14	0.047	0.98	0.94;1.05	0.555
	Clinical beds available-SUS/100,000 inhab. (July 2020)	1.09	1.03;1.15	0.007	1.02	0.98;1.08	0.343
	Clinical beds available-private system/100,000 inhab. (July 2020)	1.06	1.00;1.14	0.059	0.98	0.94;1.04	0.523
**Main exposure variables^c^**	Primary Healthcare Coverage	1.01	0.96;1.07	0.700	0.97	0.93;1.02	0.260
	Outpatient testing per case	0.98	0.93;1.03	0.401	0.98	0.93;1.03	0.318
	Distance from primary healthcare center	1.07	1.02;1.14	0.010	1.06	1.01;1.11	0.014
	Subnormal housing clusters	1.09	1.02;1.16	0.003	1.17	1.12;1.22	< 0.001
	People per bedroom (> 2)	1.22	1.15;1.30	< 0.001	1.17	1.11;1.23	< 0.001
	Proportion of non-Whites	1.15	1.08;1.22	< 0.001	1.19	1.13;1.25	< 0.001
	Literacy rate of the population aged 15 years or over	0.93	0.87;0.98	0.013	1.02	0.97;1.07	0.437
	Access to water (at least basic)	0.87	0.83;0.92	< 0.001	0.92	0.87;0.96	< 0.001
	Access to sewerage (at least basic)	0.89	0.84;0.95	< 0.001	0.88	0.84;0.93	< 0.001
	Gini index	1.03	0.97;1.09	0.395	1.11	1.06;1.16	< 0.001
	Emergency Aid	1.14	1.07;1.21	< 0.001	1.16	1.09;1.22	< 0.001

a) IRR: Incidence rate ratio; b) 95%CI: 95% confidence interval; c) Coefficients obtained with the control variables fixed in the models, for hospitalizations (diabetes mellitus hospitalization rate, respiratory diseases hospitalization rate, cardiovascular diseases mortality rate and clinical beds available/SUS per 100,000 inhab. [July de 2020]) and for mortality (diabetes mellitus mortality rate, respiratory diseases mortality rate, cardiovascular diseases mortality rate and clinical beds available/private system per 100,000 inhab. (July 2020).

**Figure 4:**
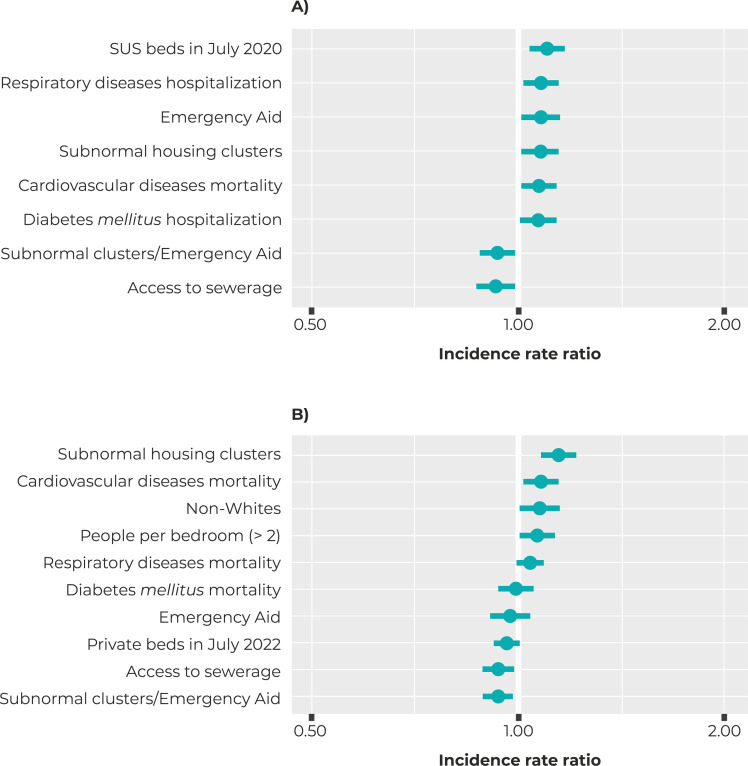
– Incidence rate ratios estimated according to a multivariable generalized linear model (GLM) for (A) COVID-19 hospitalization and (B) COVID-19 deaths, com with respective confidence intervals, in municipalities with more than 100,000 inhabitants, Brazil, 2020

The multivariable models with the best fit are shown in Figure 4. Higher COVID-19 hospitalization rates were found in municipalities with a higher proportion of the population benefiting from Emergency Aid (IRR = 1.08; 95%CI 1.01;1.16) and living in subnormal housing clusters (IRR = 1.08; 95%CI 1.01;1.16). The proportion of people with access to a sewerage system had a protective effect on COVID-19 hospitalization rates (IRR = 0.93; 95%CI 0.87;0.99). We also found higher hospitalization rates in municipalities with higher respiratory diseases hospitalization rates (IRR = 1.08; 95%CI 1.02;1.15) and diabetes *mellitus* hospitalization rates (IRR = 1.07; 95%CI 1.01;1.14), higher cardiovascular diseases mortality rates (IRR = 1.08; 95%CI 1.01;1.14), and higher number of SUS hospital beds per 100,000 inhab. in July 2020 (IRR = 1.10; 95%CI 1.05;1.16) (Figure 4a).

With regard to the COVID-19 mortality rate, positive association was found with municipalities where there was a greater proportion of non-White people (IRR = 1.08; 95%CI 1.01;1.16), a greater proportion of households in subnormal housing clusters (IRR = 1.15; 95%CI 1.09;1.21) and households with more than two people per room (IRR = 1.07; 95%CI 1.01;1.13). The proportion of people with access to a sewerage system had a protective effect on COVID-19 mortality (IRR = 0.94; 95%CI 0.90;0.99). There was a higher COVID-19 hospitalization rate in municipalities with higher respiratory disease mortality (IRR = 1.05; 95%CI 1.00;1.09) and cardiovascular disease mortality (IRR = 1.08; 95%CI 1.03;1.13) (Figure 4b).

In both the univariate and the multivariable models, interaction between the proportion of households located in subnormal housing clusters and the proportion of the population receiving Emergency Aid decreased the effect of the proportion of subnormal housing clusters (hospitalizations: IRR = 0.94; 95%CI 0.88;1.00 – deaths: IRR = 0.94; 95%CI 0.89;0.98). The two models explained, respectively, 17.0% and 14.9% of the variation in the distribution of hospitalizations and deaths without socioeconomic variables, and 23.7% and 34.7% when socioeconomic variations were considered.

The multivariate models showed no evidence of multicollinearity and were considered have a good fit, according to the deviance test, residual analysis and Cook’s distance. The variable with the highest amount of missing data was access to a sewerage system (6.4%) and the socioeconomic variables remained significant in the model, even after adjusting the data without imputation.

## DISCUSSION

The results of this study suggest that the consequences of the COVID-19 pandemic were more severe in the population with lower socioeconomic status, confirming the study hypothesis that hospital infrastructure, sex and age distribution, and presence of comorbidities are not sufficient to explain the occurrence and distribution of COVID-19 hospitalizations and COVID-19 deaths in Brazil.

The relationship found between a higher proportion of households located in subnormal housing clusters and a higher proportion of individuals living in households with more than two people per room, and the increase in COVID-19 hospitalizations and deaths, corroborates the findings that living in households located in such clusters or with more than two people per room are risk factors for the disease.^11^

Municipalities with higher respiratory disease and diabetes *mellitus* hospitalization rates and higher cardiovascular and respiratory disease mortality rates, in the period before the pandemic, had higher COVID-19 hospitalization and mortality rates, suggesting the effect of chronic diseases on COVID-19 morbidity and mortality.^6^

The ecological design of this study does not allow comparison of individual risks of illness and death due to COVID-19, but rather only enables population comparisons. Another limitation of this study is the selection of municipalities with different population sizes, which caused different variance when obtaining the estimates. To minimize this potential source of bias, municipalities with less than 100,000 inhabitants were excluded from the analysis because they have greater variance in small areas.

There may possibly be other factors, yet to be elucidated, associated with higher risk of illness and death due to COVID-19, which we have not considered here.

The strategy used to operationalize the variables, which were built from available secondary data, may also have influenced the results. To minimize the effects of this limitation, direct and indirect standardization methods were used to calculate hospitalization and mortality rates, and multiple imputation was used to estimate missing data.

Studies in different countries have shown that in addition to deaths due to COVID-19, the pandemic has contributed to an increase in deaths from other causes, especially in economically poor populations.^15^

The high transmissibility of the SARS-CoV-2 virus and local demographic characteristics influence, albeit partially, the magnitude of transmission and variations in the spread of the pandemic in different countries.^16^ Moreover, there is evidence that social and ethnic disparities are strongly related to the course of the pandemic.^17^

Type of occupation and low socioeconomic conditions may contribute to higher exposure to the virus. Despite evidence that telework is associated with lower risk of SARS-CoV-2 infection,^19^ in the case of certain types of occupation it is not possible to work from home, thus contributing to these workers being at higher risk of exposure.^20^ Moreover, in Brazil, socially disadvantaged individuals are more likely to use public transport, this being a significant risk factor for the spread of the virus: the shortcomings of this type of transport, especially seating arrangements and insufficient number of vehicles in relation to each transport route, favor crowding.^21^

As a strategy to minimize the impact of the pandemic on people’s lives and on the economy, during 2020 alone the Federal Government invested around BRL 300 billion in Emergency Aid, paid in monthly installments to the economically and socially vulnerable population.^22^ Our analysis showed that the proportion of the population that received Emergency Aid was related to the decrease in the effect of the proportion of subnormal housing clusters on the increase in COVID-19 hospitalization and mortality rates. This result emphasizes that Emergency Aid contributed to minimizing the impacts of COVID-19 on the population.

Around 80% of the Brazilian population depends exclusively on the SUS for any kind of health care.^23^ As such, the COVID-19 pandemic was one of the biggest challenges faced by the public health system since its creation, due to the need to overcome local and regional inequalities in the provision of care to COVID-19 cases, ensure more hospital beds, purchase equipment and supplies in record time, in addition to the need for strategies to promote articulation between public and private health services, with a view to access and quality of care provided to the population. The concentration of specialized health services in medium-sized cities and state capitals impacted the logistical organization of resources and the composition of medical teams, requiring critically ill patients to be transported to these centers and, consequently, affecting the care they received.^24^

Corroborating data from a previous study, our analysis showed that populations living in areas distant from the primary health care network were at higher risk of hospitalization. A cohort study conducted in Scotland showed that individuals living in socioeconomically disadvantaged areas had lower hospitalization rates and worse outcomes after intensive care. That study also showed that the demand for beds at health centers located in the most economically disadvantaged areas was greater than available bed capacity.^25^

In our study, municipalities that had a higher proportion of non-White population and/or people living in subnormal housing clusters, and those with a higher proportion of households with more than two people per room, had higher COVID-19 mortality rates. These findings corroborate data in the literature indicating ethnic, racial and socioeconomic discrepancies in COVID-19 mortality.^26^

The proportion of access to a sewerage system was a protective factor for COVID-19 mortality. Scarcity of sewerage systems is associated with poverty, affecting the health conditions of the population, in addition to other factors such as malnutrition and inadequate hygiene.^29^

As in our study, Li et al.^30^ also found that non-White people were at greater risk of dying from COVID-19 in São Paulo, Brazil. Several factors contributed to the increased risks faced by non-White people, including: greater risk of exposure to the virus; greater risk of infection after exposure; poorer health status and greater susceptibility to severe diseases; not seeking health care adequately; and poorer quality of health care received.^28^ Undeniably, racial and socioeconomic inequalities continue to have persistent, significant and multifaceted associations with health problems, thus reproducing historical patterns.

This study showed that socioeconomic inequality influenced the distribution of COVID-19 in Brazil’s largest municipalities. Despite the importance of indicators such as hospital infrastructure and comorbidity prevalence, the place and conditions in which people live, their ethnicity, and their access to health care and social policies, especially in times of crisis, had an important impact on the course of the pandemic in Brazil.

Finally, we recommend that further studies be conducted on the social determinants of COVID-19 in Brazil, with a special focus on the role of income redistribution programs. A better understanding of the phenomenon will be important to inform the planning of future social programs, including in contexts of social calamity, as happened during the course of the COVID-19 pandemic.
